# Albumin-neprilysin fusion protein: understanding stability using small angle X-ray scattering and molecular dynamic simulations

**DOI:** 10.1038/s41598-020-67002-9

**Published:** 2020-06-22

**Authors:** Alina Kulakova, Sowmya Indrakumar, Pernille Sønderby Tuelung, Sujata Mahapatra, Werner W. Streicher, Günther H. J. Peters, Pernille Harris

**Affiliations:** 1 Department of Chemistry, Technical University of Denmark, Kemitorvet building 207, Kgs. Lyngby, 2800 Denmark; 2 Novozymes A/S, Biologiens Vej 2, 2800 Kgs. Lyngby, Denmark

**Keywords:** Molecular modelling, SAXS, High-throughput screening

## Abstract

Fusion technology is widely used in protein-drug development to increase activity, stability, and bioavailability of protein therapeutics. Fusion proteins, like any other type of biopharmaceuticals, need to remain stable during production and storage. Due to the high complexity and additional intramolecular interactions, it is not possible to predict the behavior of fusion proteins based on the behavior the individual proteins. Therefore, understanding the stability of fusion proteins on the molecular level is crucial for the development of biopharmaceuticals. The current study on the albumin-neprilysin (HSA-NEP) fusion protein uses a combination of thermal and chemical unfolding with small angle X-ray scattering and molecular dynamics simulations to show a correlation between decreasing stability and increasing repulsive interactions, which is unusual for most biopharmaceuticals. It is also seen that HSA-NEP is not fully flexible: it is present in both compact and extended conformations. Additionally, the volume fraction of each conformation changes with pH. Finally, the presence of NaCl and arginine increases stability at pH 6.5, but decreases stability at pH 5.0.

## Introduction

Stability and efficacy of protein-drugs are essential to achieve desirable biopharmaceutical applications. One of the strategies in optimization of protein-therapeutics is fusion technology, which consists of linking a target protein to a more stable protein. This approach has shown to improve catalytic efficiency, activity, stability, and solubility of protein-drugs^1^. Moreover, the fusion-protein approach is used to prevent fast renal clearance by connecting a target protein to a protein with a longer half-life. Human serum albumin (HSA), which is used in this study, and immunoglobulin Gs are proteins widely used in fusion technology due to their long half-life.

HSA is a highly abundant and well-studied serum protein with a half-life around 19–22 days^2^. Currently, multiple albumin fusion proteins are under clinical trials, and two are already accepted by the Food and Drug Administration (FDA)^3^. One of them is albiglutide: an albumin fusion protein connected to a glucagon like peptide-1 receptor agonist^4^. This therapeutic is administered for treatment of type 2 diabetes. The second album fusion drug is albutrepenonacog alfa, linked to the recombinant coagulation factor IX, which is used for treatment of hemophilia B^5^. In both therapeutics, the presence of HSA contributes to a significant increase in half-life: from 1.5–5 min^6^ up to 3.6–8 days for albiglutide, and from 17–34 h up to 92 h for albutrepenonacog alfa^5,7^.

As mentioned above, fusion technology can be used to address a variety of problems in protein-drug development. However, as any other type of biopharmaceutical, fusion proteins require special conditions (formulation) that will preserve their stability during production and storage. As it is not yet possible to predict the behavior of different proteins under different conditions, formulation remains a long and expensive process in protein-drug production. Formulation is particularly challenging for fusion proteins^7^, as additional intramolecular interactions lead to a change in stability, and therefore it is hard to predict the behavior of fusion proteins based on stability of individual proteins.

**Figure 1:**
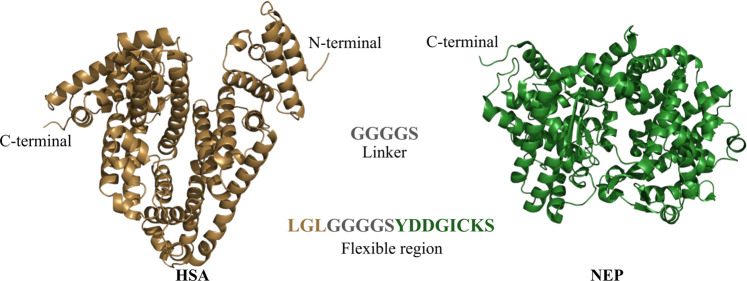
HSA N-terminus fused with the NEP C-terminus via GGGGS linker (figure created using *PyMOL*^25^).

The aim of this study is to provide a better understanding on fusion protein stability and relate it to conformational changes. Specifically, we are investigating the stability of albumin fused to neprilisyn (HSA-NEP) (see Fig. 1). NEP is widely distributed in mammalian tissues and is involved in the inactivation of a variety of signaling peptides^8,9^. Additionally, it is involved in the degradation of amyloid β peptides, which makes it an attractive candidate as a protein-drug for treatment of Alzheimer’s disease^10^.

HSA-NEP was studied together with other proteins in PIPPI library^11^, and it was clear that the behavior of this protein was different from other flexible proteins, such as monoclonal antibodies.

In this study, the overall stability of HSA-NEP was investigated by thermal and chemical denaturation by varying pH and buffer composition, complemented by small angle X-ray scattering (SAXS) and molecular dynamics (MD) simulations. In combination, the stability studies and SAXS show a correlation between increasing protein repulsion and decreasing conformational stability. This contradicts conclusions from a previous study on a similar fusion protein: albumin fused with human growth hormone (HSA-hGH)^12^, where repulsion is concluded to have a stabilizing effect at certain conditions. Additionally, combining SAXS results with MD simulation results provided a molecular understanding of the determinants that cause the HSA-NEP conformational changes.

## Results

The overall stability of HSA-NEP was analyzed by thermal and chemical denaturation using nano differential scanning fluorimetry (nanoDSF) and isothermal chemical denaturation (ICD). Initially, ICD was performed using two different denaturants: urea and guanidine hydrochloride (GuHCl). GuHCl is a strong denaturant that starts to unfold HSA-NEP at low concentrations. Therefore, urea was used for subsequent studies, in order to obtain well-defined denaturation curves. The initial analysis was performed as a function of pH (5–9) and NaCl concentration (0, 70, and 140 mM), reaching ionic strength of 150 mM. In this way, pH and ionic strength range covered most of the formulation conditions of biopharmaceuticals.

### pH dependence

**Figure 2:**
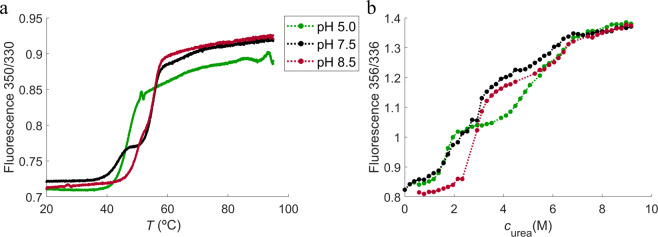
NanoDSF (**a**) and ICD (**b**) curves at 10 mM histidine pH 5.0 (green), 10 mM histidine pH 7.5 (black), and 10 mM tris pH 8.5 (red).

The denaturation curves from thermal unfolding using nanoDSF are shown in Fig. 2a. HSA-NEP has a single two-state unfolding (from folded to unfolded state) at pH 5.0, which is shifted towards a three-state unfolding with increasing pH (with the presence of the intermediate state). However, from pH 7.5 to pH 8.5 the thermal unfolding shifts back to a two-state (see Fig. 2a). Chemical denaturation results in a multi-state unfolding (with two intermediate states), which is shifted towards a three-state unfolding with increasing pH (see Fig. 2b). This means that the first intermediate state has a clear plateau at pH 5.0, less clear at pH 7.5, and not apparent at pH 8.5. The second intermediate state is not well-defined at pH 5.0, but more pronounced at pH 7.5 and 8.5. Hence, thermal and chemical denaturation of HSA-NEP show different unfolding mechanisms. It is difficult to speculate why, but one of the possible explanations is that the presence of urea in chemical denaturation inhibits aggregation, while thermal denaturation does not.

**Figure 3:**
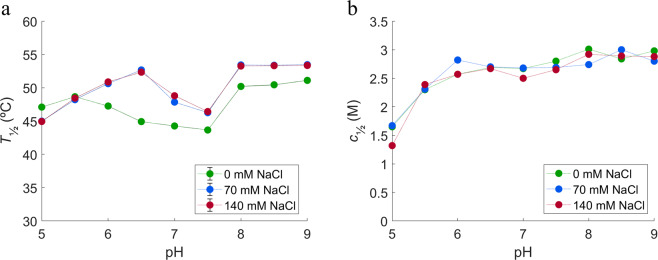
Initial stability studies performed using (**a**) NanoDSF and (**b**) ICD at different pH and ionic strengths. In green: 0 mM NaCl, in blue: 70 mM NaCl, and in red: 140 mM NaCl.

The temperature of unfolding (*T*_½_) and the denaturant needed to unfold 50% of the protein (*c*_½_) are shown in the Fig. 3. At 0 mM NaCl, *T*_½_ decreases from pH 5.5 to 7.5, with the higher values at lower (pH 5.0 and 5.5) and higher pH (from pH 8.0 to pH 9.0) (see Fig. 3a). The *c*_½_ increases from pH 5.0 to pH 6.0, where it reaches a plateau. It increases again around pH 8.0 (see Fig. 3b).

**Figure 4:**
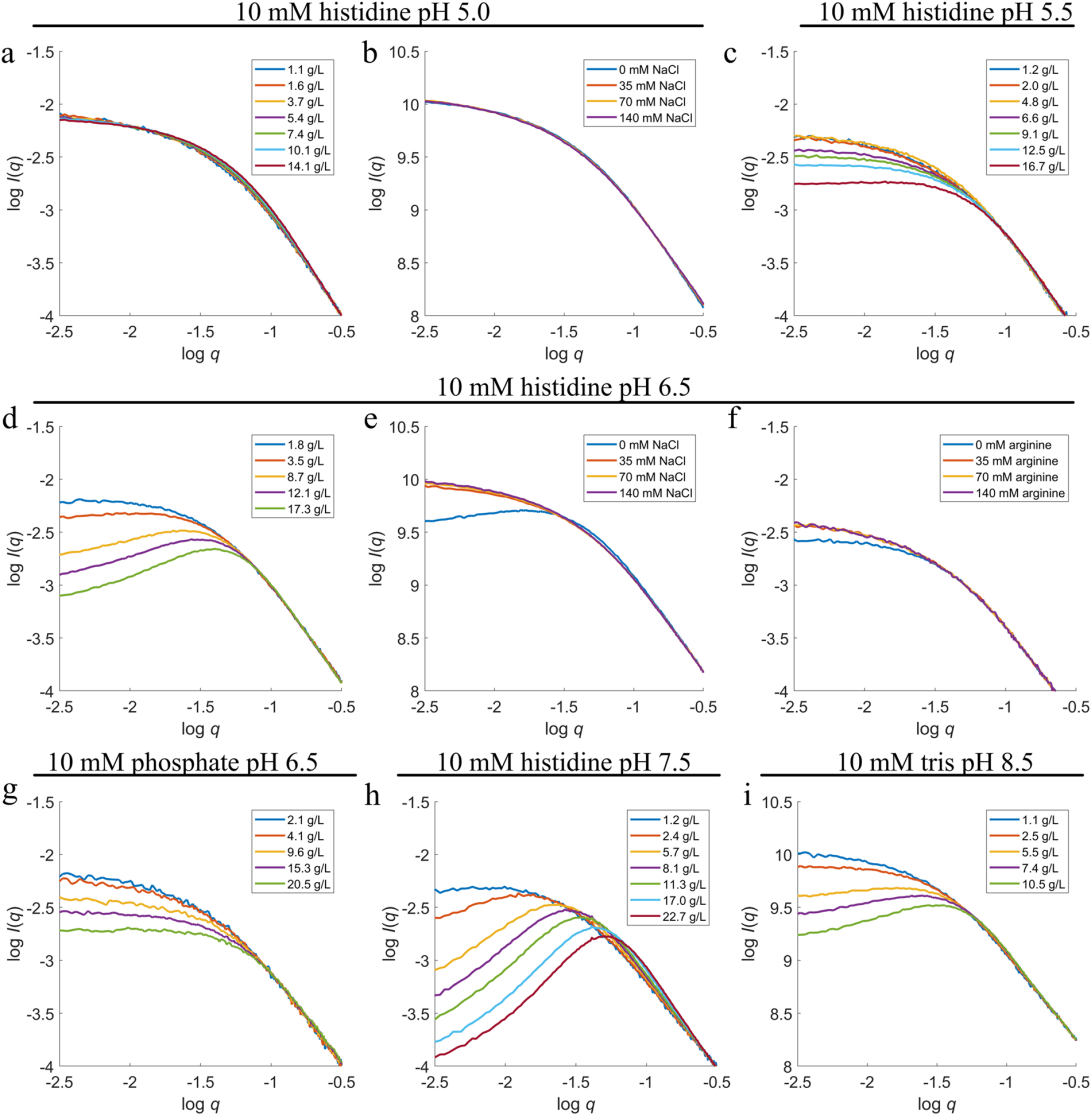
SAXS scattering curves for concentration series at (**a**) 10 mM histidine pH 5.0; (**c**) 10 mM histidine pH 5.5; (**d**) 10 mM histidine pH 6.5; (**g**) 10 mM phosphate pH 6.5; (**h**) 10 mM histidine pH 7.5; (**i**) 10 mM tris pH 8.5. SAXS scattering curves varying *c*_NaCl_ at (**b**) 10 mM histidine pH 5.0 and (**e**) 10 mM histidine pH 6.5, with *c*_HSA-NEP_ around 5.5–6 g/L; and varying *c*_arginine_ at (**f**) 10 mM histidine pH 6.5 with *c*_HSA-NEP_ around 2 g/L.

In order to study the associated conformational changes, SAXS concentration series data were collected at 10 mM histidine at pH 5.0, 5.5, 6.5, and 7.5, and 10 mM tris at pH 8.5 with 0 mM NaCl. All scattering curves and SAXS data analysis can be seen in supplementary information (SI) (see Table S.2 and Figure S.1). The intensity at low *q*-values decreases with increasing HSA-NEP concentration at all pH values (except pH 5.0) indicating a repulsive system (see Fig. 4). Moreover, repulsive interactions increase from pH 5.5 to 7.5 and decrease from pH 7.5 to 8.5, which correlates with the observed changes in *T*_½_ (see Fig. 3a).

**Figure 5:**
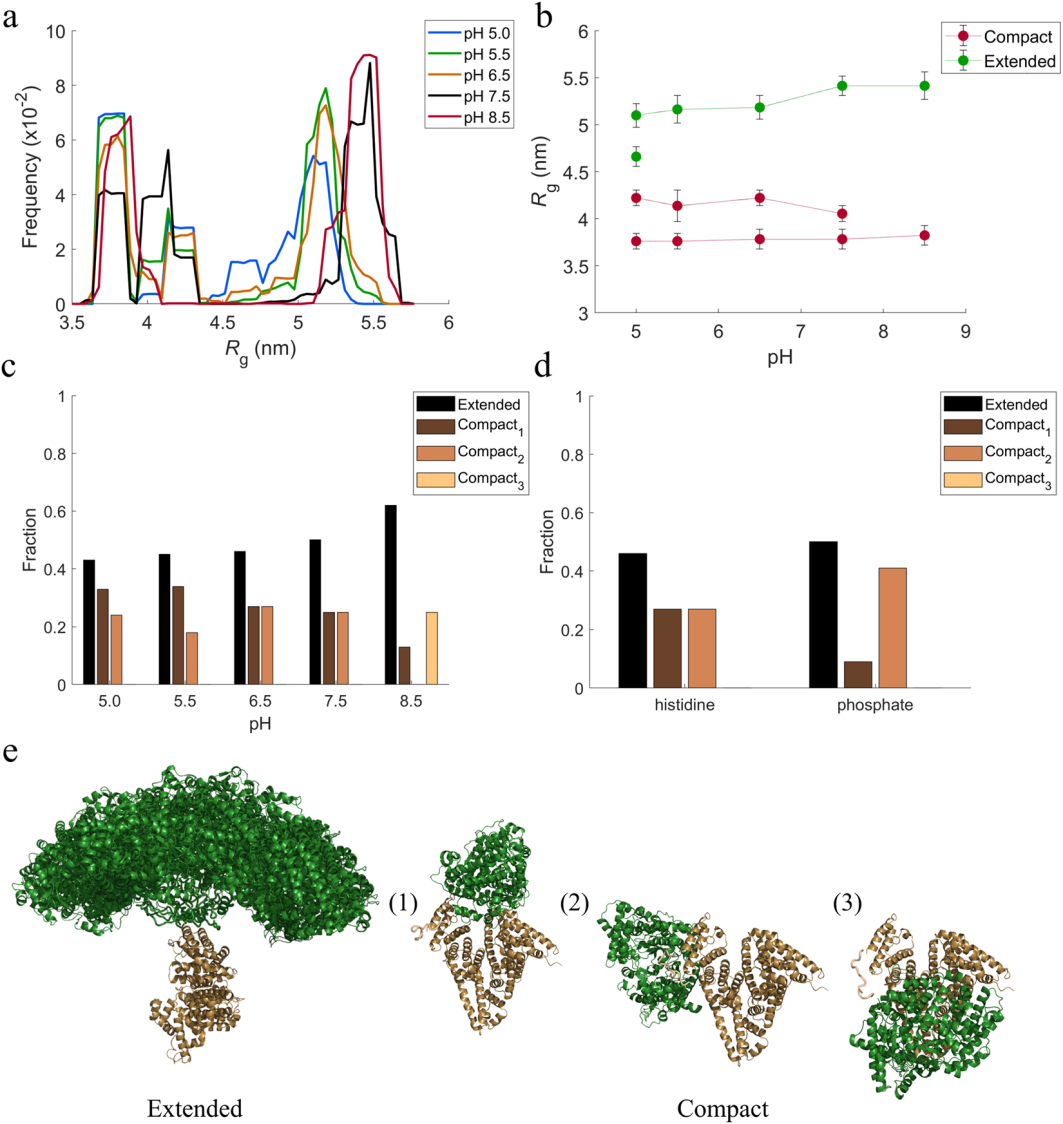
Analysis from *EOM*. (**a,b**) *R*_g_ distributions, (**c**) volume fractions of different conformations in 10 mM histidine at pH 5.0, 5.5, 6.5, and 7.5 and 10 mM tris pH 8.5, (**d**) volume fractions of different conformations at pH 6.5 in histidine and phosphate buffers. (**e**) high resolution models for extended and compact conformations (figures created using *PyMOL*^25^).

The Kratky plots of HSA-NEP show increase of the scattering at higher angles, which is characteristic for flexible systems^13^ (see Fig. S.2 in SI). Therefore, conformational changes of HSA-NEP were studied using *Ensemble Optimization Method* (*EOM*)^14,15^, which accounts for flexibility. This means that the relative position of HSA and NEP was allowed to vary under the constrains of the linker. HSA (PDB ID: 6EZQ^16^) and NEP (PDB ID: 6GID^17^) crystal structures were used as template structures for HSA and NEP. The *EOM* results were analyzed by looking at the resulting high-resolution structures, their probability (volume fractions) and radius of gyration (*R*_g_) distribution. At all the investigated pH values, the *R*_g_ distributions shown in Fig. 5a,b, indicate the presence of two overall populations: a more compact conformation with *R*_g_ around 4 nm and a more extended conformation with *R*_g_ around 5 nm.

The *R*_g_ distribution around 4 nm shows multiple peaks, indicating the presence of multiple compact conformations. The detailed SAXS analysis of the high-resolution models shows the presence of three different compact conformations (see Fig. 5e), whose distribution is pH dependent (see Fig. 5c).

The volume fraction of the extended conformation, as well as *R*_g_, increase with increasing pH. The analysis of the high-resolution models shows many different possible conformations, which is an indication of high flexibility of the extended conformation. This is illustrated in Fig. 5e, where HSA is kept fixed, and the position of NEP is seen to vary amongst the suggested structures.

### NaCl dependence

With the addition of NaCl, the thermal denaturation studies show an increase in *T*_½_ above pH 5.5 which points to an increase in thermal stability (see Fig. 3a), while ICD studies do not show a clear trend for NaCl effect. The SAXS experiments were performed in the presence of 0, 35, 70, and 140 mM NaCl at 10 mM histidine pH 5.0 and 6.5 (see Fig. 4b,e). At pH 5.0, the scattering curves do not change with the presence of NaCl. However, at pH 6.5 the intensity at low *q*-values increases with addition of NaCl and calculated molecular weight (*MW*) shifts from 136 to 150 kDa (see Table S.3 in SI), which is closer to the real *MW* of HSA-NEP. These results show that addition of NaCl screens the repulsive interactions present at pH 6.5, 0 mM NaCl.

### Buffer and excipients dependence

**Figure 6:**
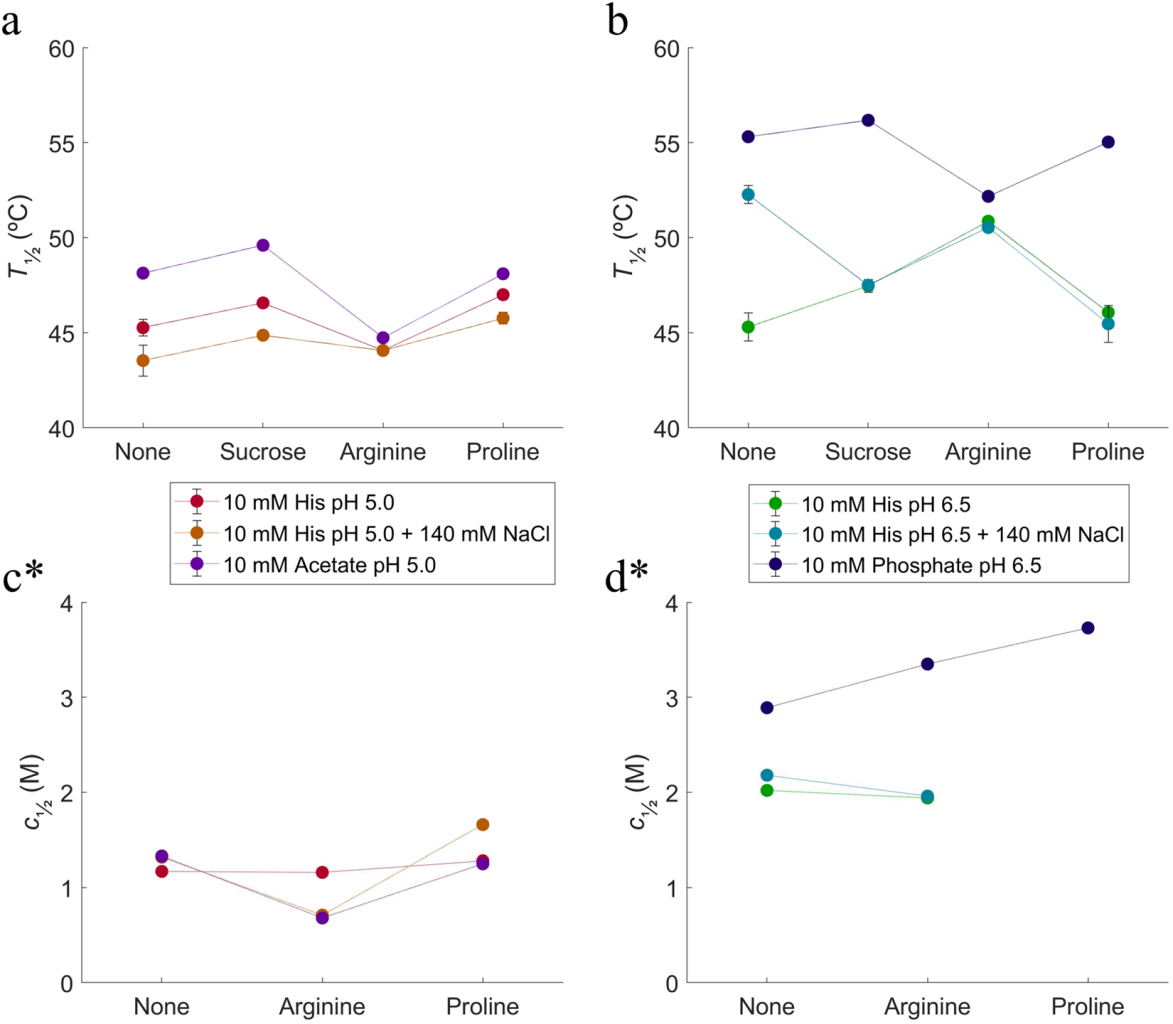
NanoDSF and ICD stability studies using different buffers and excipients. (**a**) changes in *T*_½_ at histidine (0 and 140 mM NaCl) and acetate pH 5.0; (**b**) changes in *T*_½_ at histidine (0 and 140 mM NaCl) and phosphate pH 6.5; (**c**) changes in *c*_½_ at histidine (0 and 140 mM NaCl) and acetate pH 5.0; (**d**) changes in *c*_½_ at histidine (0 and 140 mM NaCl) and phosphate pH 6.5. Purple: 10 mM acetate pH 5.0, red: 10 mM histidine pH 5.0, orange: 10 mM histidine pH 5.0 with 140 mM NaCl, blue: 10 mM phosphate pH 6.5, green: 10 mM histidine pH 6.5, cyan: 10 mM histidine pH 6.5 with 140 mM NaCl. *It was not possible to perform ICD studies at pH 5.0 with sucrose and pH 6.5 with sucrose and proline, due to the crystallization of solution in high concentrations of urea.

Relative to the histidine buffer, both the acetate and phosphate buffers give rise to higher *T*_½_ and *c*_½_ (see Fig. 6), which points to a higher conformational stability of the protein. Sucrose, arginine, and proline were selected as excipients and tested in the different buffers.

At pH 5.0, both sucrose and proline have a weak positive effect on the HSA-NEP thermal stability, while the effect of arginine is negative and more pronounced. In acetate buffer, the presence of arginine is destabilizing, as it causes a decrease in *T*_½_ and *c*_½_ (see Fig. 6a,c, and Table 1). In histidine pH 5.0, arginine does not have a significant effect on the thermal stability, but the ICD studies show a decrease in *c*_½_ by around 1 M in the presence of NaCl.

**Table 1:** Overview of the effect of excipients deduced from nanoDSF (*T*_½_) and ICD (*c*_½_) data analyses. Suc: sucrose, Arg: arginine-HCl, Pro: proline.

	10 mM acetate pH 5.0			10 mM histidine pH 5.0					
							+140 mM NaCl		
	Suc	Arg	Pro	Suc	Arg	Pro	Suc	Arg	Pro
*T*_½_	+	−−	0	+	−	+	+	+	++
*c*_½_	+	−−	0	0	0	0	x	−−	+
	**10 mM phosphate pH 6.5**			**10 mM histidine pH 6.5**					
							**+140 mM NaCl**		
	**Suc**	**Arg**	**Pro**	**Suc**	**Arg**	**Pro**	**Suc**	**Arg**	**Pro**
*T*_½_	+	−−	−	++	+++	+	−−	−	−−−
*c*_½_	x	+	++	x	0	x	x	−	X

++++/−−−− Δ*T*_½_ > 10 °C Δ*c*_½_ > 1.5 M.

+++/−−−5 °C < Δ*T*_½_ < 10 °C 1 M < Δ*c*_½_ < 1.5 M.

++/−−2 °C < Δ*T*_½_ < 5 °C 0.5 M < Δ*c*_½_ < 1.0 M.

+/− 0.5 °C < Δ*T*_½_ < 2 °C 0.2 M < Δ*c*_½_ < 0.5 M.

0 Δ*T*_½_ < 0.5 °C Δ*c*_½_ < 0.2 M.

Reference point for excipients: respective buffer without excipient.

+ stabilizes.

− destabilizes.

x – data not acquired.

The phosphate buffer at pH 6.5 was selected for SAXS measurements, as it has a clear positive effect in both ICD and nanoDSF experiments (see Fig. 6b,d and Table 1). Like in the histidine buffer, the intensity curves are decreasing at low *q*-values, with increasing *c*_HSA-NEP_ (see Fig. 4d,g), which points to the presence of repulsive interactions. However, this decrease is less pronounced in phosphate buffer, which means that the system is less repulsive. At ~3 g/L, the calculated *MW*_HSA-NEP_ is lower in phosphate buffer (166 kDa) than in histidine buffer (180 kDa) (see Table S.3 in SI), which points to a lower amount of larger species/aggregates in phosphate buffer. Moreover, the volume fractions of the compact conformations differs significantly: in histidine buffer both conformations, compact_1_ and compact_2_, are present in equal amounts (0.27), while in phosphate buffer, the amount of compact_1_ has decreased to 0.13 and the amount of compact_2_ has increased to 0.41 (see Fig. 5d).

At pH 6.5, the three excipients affect the system differently. Sucrose is slightly stabilizing in phosphate and histidine buffers, but in combination with NaCl it has a destabilizing effect, decreasing *T*_½_ by 4.8 °C (see Fig. 6b and Table 1). Proline has some stabilizing effect in histidine buffer, but in combination with NaCl it decreases *T*_½_ by 6.8 °C, which points to a decrease in stability. In phosphate buffer, both proline and arginine have a different effect in thermal and chemical denaturation studies: they have negative effect on thermal stability, but seem to protect HSA-NEP from chemical denaturation (see Fig. 6b,d, and Table 1). In histidine buffer at pH 6.5 arginine increases *T*_½_ by 5.4 °C, which means an increase in HSA-NEP stability. Additionally, SAXS data show a decrease in the intermolecular repulsion (see Fig. 4f). Addition of arginine in combination with NaCl has no effect.

### Molecular dynamics simulations

In order to understand conformational changes of HSA-NEP under different physicochemical conditions, the interface between HSA and NEP in extended and compact conformations were studied using MD simulations, using high-resolution models based on SAXS data generated by *EOM*. Subsequently, changes in the electrostatic surface potentials at the protein-protein interface were investigated to understand conformational preference with pH.

### Extended

**Figure 7:**
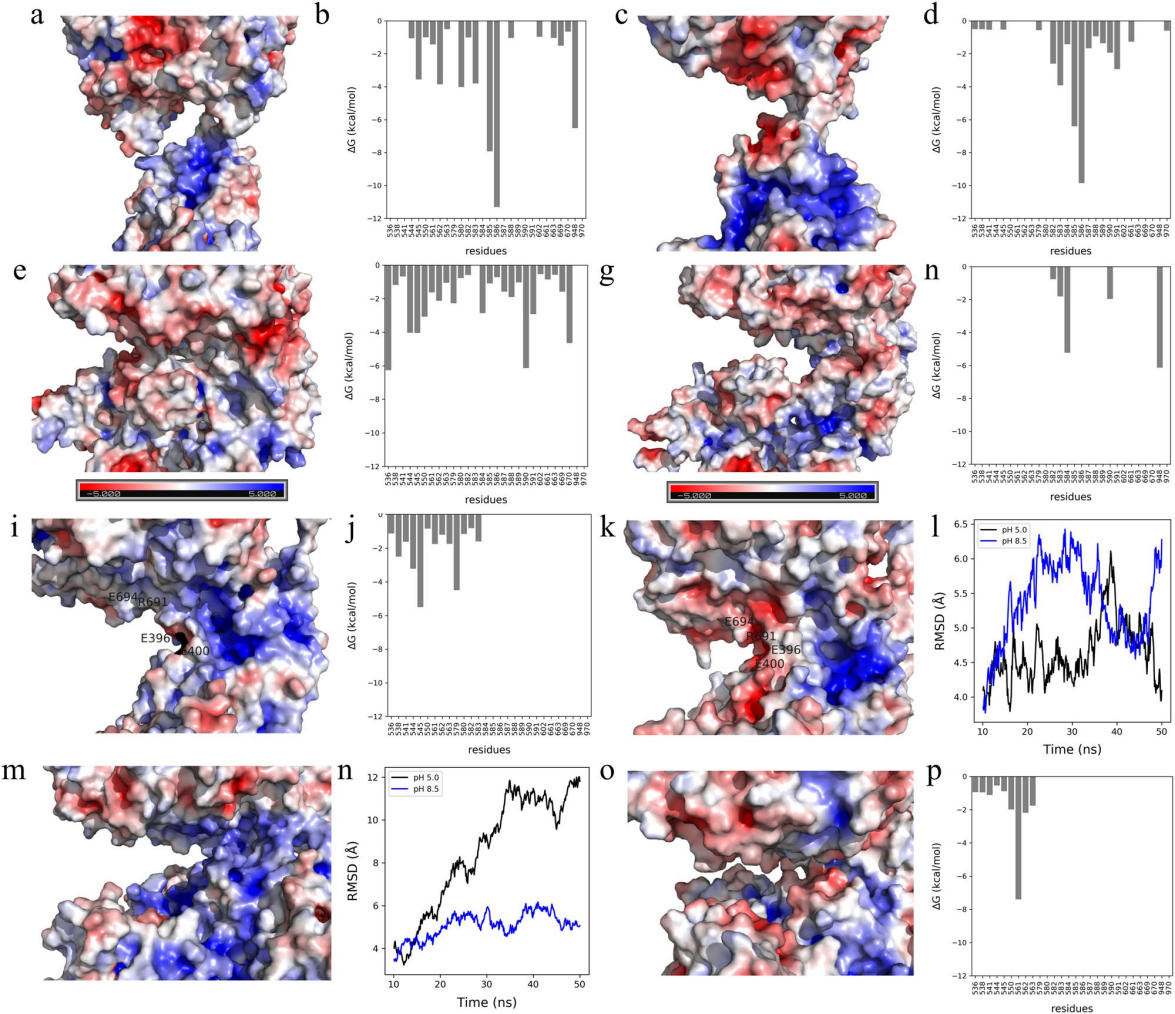
Surface coloring of the representative structure from MD trajectory clustering based on the electrostatic potentials at pH 5.0 for (**a**) extended conformation, (**e**) compact_1_, (**i**) compact_2_, and (**m**) compact_3_; at pH 8.5 for (**c**) extended conformation, (**g**) compact_1_, (**k**) compact_2_, and (**o**) compact_3_ (color-scale: red, white, and blue indicates negative, close to neutral, and positive potentials. Figures were created using *PyMOL*^25^). HSA is located in the bottom and NEP in the top. Interaction energy at pH 5.0 for (**b**) extended conformation, (**f**) compact_1_, and (**j**) compact_2_; at pH 8.5 for (**d**) extended conformation, (**h**) compact_1_ and (**p**) compact_3_. RMSD plot for (**l**) compact_2_, and (**n**) compact_3_ at pH 5.0 (in black) and 8.5 (in blue).

The electrostatic surface and interaction energy for inter-domain interface of the extended conformation are shown in Fig. 7a–d. The electrostatic surface at pH 5.0 shows less prominent positive and negative patches when compared to pH 8.5 (see Fig. 7a,c). Furthermore, the free energy of interaction at the interface region is similar as they do not share a large interface in extended conformation. Due to the neutral patches around the interface, HSA and NEP tend to be closer in space at pH 5.0 (*R*_g_ of 45.97 ± 0.22 Å). Contrary to this, both proteins remain far from each other at pH 8.5 (*R*_g_ of 50.20 ± 0.73 Å), due to repulsive negative charge-charge interactions.

### Compact_1_

The electrostatic surface and interaction energy for compact_1_ inter-domain interface are shown in Fig. 7e–h. With increasing pH, residues at the interface have a lower contribution to the free energy of interaction (see Fig. 7f,h). This is in agreement with the analysis of intermolecular interactions, which points to the presence of three salt bridges (E505-R665, D563-K665, and K564-D659) and two hydrogen bonds (R114-R633 and N503-Q610) at pH 5.0, and only one salt bridge (E565-K665) at pH 8.5. This change in the interactions from pH 5.0 to 8.5 leads to increase in *R*_g_ from 37.58 (±0.23 Å) to 39.67 (±0.48 Å) resulting in less compact conformation.

### Compact_2_

The electrostatic surface and interaction energy for compact_2_ inter-domain interface are shown in Fig. 7i–k. In the compact_2_ conformation, the electrostatics change with pH around the interface region, which include residues E694, R691, D1044, E396, and E400 (see Fig. 7i,k). With increasing pH, the interface region becomes more hydrophilic, resulting in negative charge-charge repulsion. Moreover, solvent accessible surface area (SASA) plots as a function of simulation time shows that compact_2_ has increasing interface SASA with increasing pH (data not shown). Additionally, the root mean square deviation (RMSD) shows that compact_2_ is unstable at pH 8.5 (see Fig. 7l).

### Compact_3_

The electrostatic surface and interaction energy for compact_3_ inter-domain interface are shown in Fig. 7m–p. At pH 5.0, compact_3_ conformation has a strong positive patch in the interface region, which makes HSA and NEP prone to repulsive behavior (see Fig. 7m). With increasing pH, the interface becomes neutral (see Fig. 7o), which makes compact_3_ conformation more favorable at pH 8.5. This is in agreement with analysis of intermolecular interactions: at pH 5.0 there are no interactions predicted, while at pH 8.5 they are three hydrogen bonds (K475-N634, D494-Q610, and E495-Q610). Additionally, SASA of the interface increases (100 Å) with decreasing pH and the RMSD shows that compact_3_ is unstable at pH 5.0 (see Fig. 7n). The overall interface energy at pH 8.5 favors compact_3_ in tris buffer, decreasing from −17.8 to −24.0 kCal/mol.

### Denaturation process

**Figure 8:**
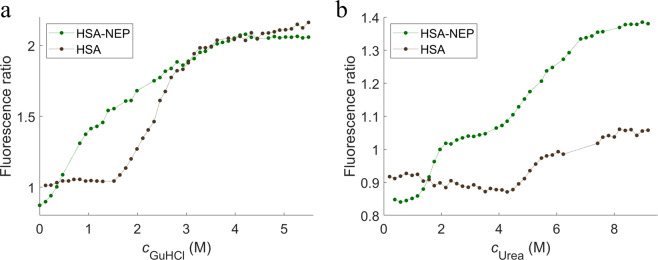
Chemical unfolding curves for HSA-NEP (in green) and HSA (in black) in histidine at pH 5.0 with (**a**) GuHCl and (**b**) urea.

Both urea and GuHCl were used as denaturants in the chemical denaturation studies, and both show a multi-state unfolding (see Fig. 8a,b). However, they point to different unfolding mechanisms: in the presence of urea, the first two transitions are well separated with well-defined intermediate states, which is not the case with GuHCl. Unlike HSA-NEP, HSA alone has a simple two-state unfolding mechanism and requires higher concentrations of urea (~5 M) and GuHCl (~2.3 M) to unfold. It is seen in Fig. 8b that HSA alone starts to unfold with addition of around 4 M urea, which corresponds to the beginning of the second transition of HSA-NEP. This suggests that in the presence of urea, the NEP domain unfolds first.

**Figure 9:**
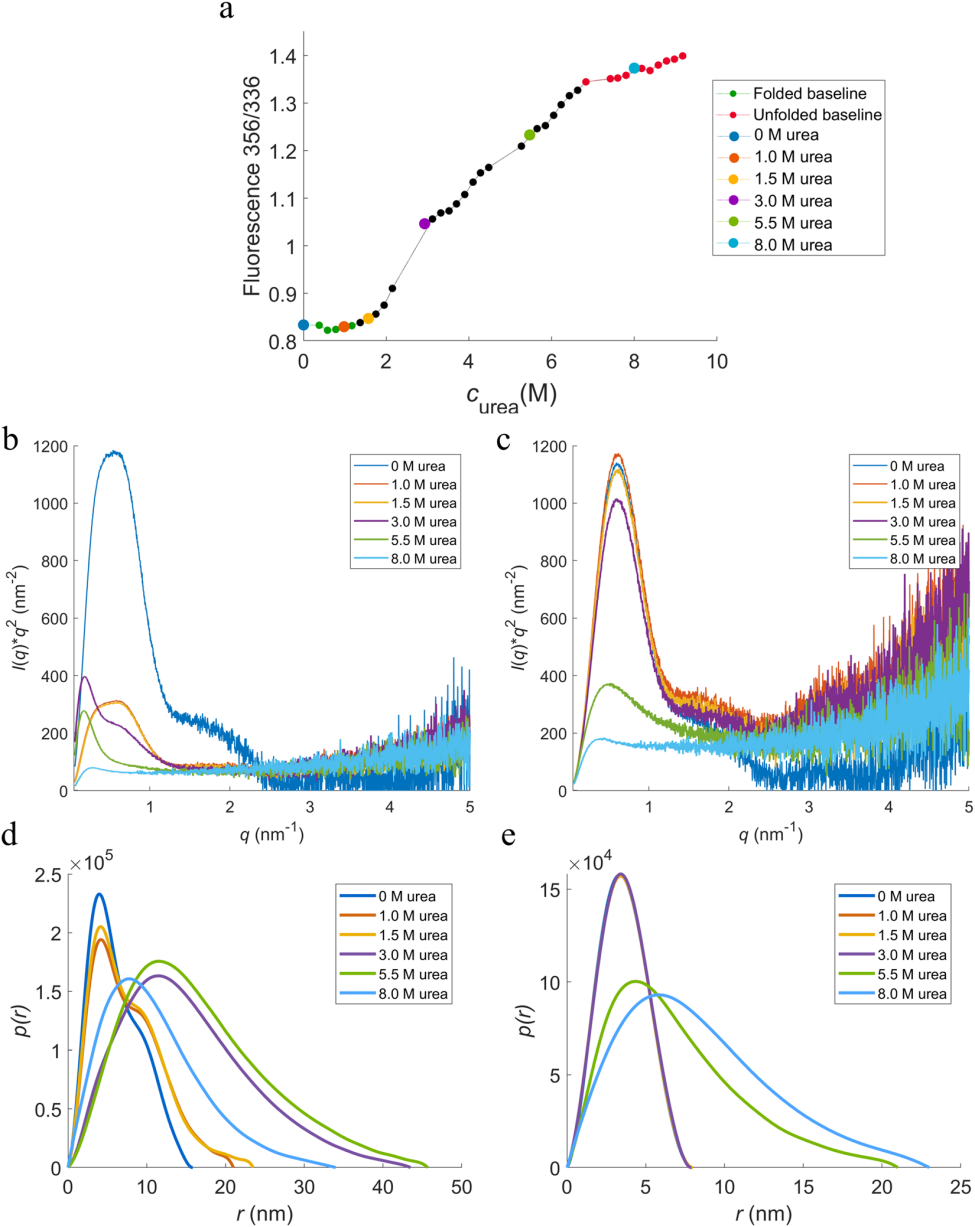
HSA-NEP chemical denaturation study. (**a**) HSA-NEP (1 g/L) chemical unfolding curve. Kratky plots for (**b**) HSA-NEP (5 g/L) at different concentrations of urea, and for (**c**) HSA (5 g/L) at different concentrations of urea. *p*(r) functions for (**d**) HSA-NEP (5 g/L) at different concentrations of urea, and for (**e**) HSA (5 g/L) at different concentrations of urea. Urea concentrations are given in the insets.

In order to confirm the previous statement and follow conformational changes during unfolding, SAXS experiments were performed at different concentrations of urea in 10 mM histidine at pH 5.5, where the transitions are more well-defined (see Fig. 9a,b). Without denaturant, HSA-NEP has a Kratky plot with a well-defined maximum and pair distribution (*p*(*r*)) function with a double peak, which is characteristic for multidomain proteins. In the presence of 1 and 1.5 M of urea, the shape of the peak changes, pointing to small conformational changes. By increasing the concentration up to 3 M, the shape of the peak shifts to a single peak, which indicates significant conformational changes. The maximum intensity in the Kratky plot decreases with increasing concentration of urea, due to decreasing contrast. Finally, in the presence of 8 M urea, the Kratky plot has a plateau instead of a peak, meaning that HSA-NEP is fully unfolded.

The same experiment was performed with HSA alone (see Fig. 9c). With addition of 1, 1.5, and 3 M urea, the shape of the peak in *p*(*r*) function remains the same. By adding 5.5 M urea the peak becomes broader and the intensity increases at high *q*-values, which is characteristic for partially unfolded proteins. At the maximum concentration of urea, the Kratky plot of HSA (as well as HSA-NEP) has a plateau instead of a peak, which is characteristic of a fully unfolded protein.

## Discussion

**Figure 10:**
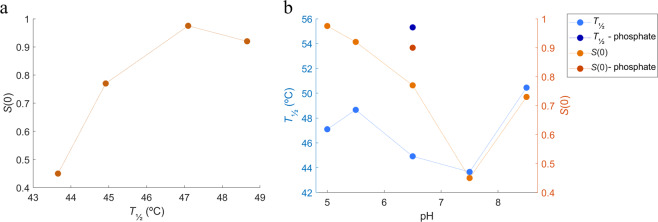
Correlation between thermal stability and repulsive interactions with pH. (**a**) correlation between *S*_0_ and *T*_½_, (**b**) correlation between structure factor and *T*_½_ in histidine buffer (pH 5.0–7.5), phosphate buffer (pH 6.5), and tris buffer (pH 8.5). Thermal stability represented as *T*_½_ (in blue); and repulsive interactions represented as structure factors at *c*_HSA-NEP_ around 10 g/L (in orange).

From the nanoDSF and SAXS data analyses it is clear that there seem to be a correlation between *T*_½_ and repulsive interactions in the system (see Fig. 10a,b). An increase in repulsion, seen as a decrease in the structure factor, *S*(0), in the SAXS data, is followed by a decrease in *T*_½_, which could be indicative of a destabilizing effect of repulsive interactions. The correlation seems to be dependent also on the choice of buffer, as seen in Fig. 10b. This finding contradicts the findings of *Cordes et al*.^12^, where repulsive interactions in 10 mM acetate at pH 5.0 show a positive effect on the HSA-hGH fusion protein stability^12^. Due to the similar pKa values for hGH and NEP (5.12 for hGH^18^ and 5.47 for NEP), similar non-specific protein-protein interactions would be expected, however the specific interactions must be responsible for these differences.

The increase in repulsion seen in histidine buffer from pH 5.5 to 7.5 can be explained by the pI values of the individual proteins. HSA has pI of 5.67 and NEP has pI of 5.47, which means that both proteins are negatively charged at pH> 6. Therefore, increasing pH leads to an increase in repulsive interactions, which is also in agreement with MD simulations (see Fig. 7). The increase in repulsion between the artificially connected HSA and NEP proteins could induce internal stress, which, according to the SAXS data, is followed by an increased volume fraction of the extended conformation. One possible scenario is that in order to minimize the internal repulsive interactions in the protein, both domains keep as far away from each other as possible, which leads to an increase in *R*_g_ (see Fig. 5a,b). This results in increased flexibility and that HSA-NEP is more easily unfolded. Additionally, from two possible conformations, HSA-NEP will prefer the extended conformation, where HSA and NEP are more separated in space (see Fig. 5c).

Furthermore, it is seen that at pH 6.5, where the protein clearly shows repulsive interactions and low thermal stability, the addition of NaCl to HSA-NEP increases *T*_½_ (see Fig. 3a). At the same time, SAXS data indicates that NaCl screens the repulsive interactions (see Fig. 4e), leading to increase in stability.

NanoDSF and ICD studies show that not only pH but also the buffer type influences HSA-NEP stability. Changing histidine buffer for phosphate buffer at pH 6.5 increases both *T*_½_ and *c*_½_ (see Fig. 6b,d), decreases repulsion (see Fig. 4e,g), and changes the volume fractions of compact_1_ and compact_2_ (see Fig. 5d). One of the reasons might be the significantly higher ionic strength in 10 mM phosphate buffer at pH 6.5 (0.013 M vs 0.005 M for 10 mM histidine buffer), which contributes to the screening of repulsive interactions. The changes in volume factions for the compact conformations might happen due to specific interactions between the phosphate buffer and HSA-NEP. At pH 5.0, HSA and NEP do not have significant intramolecular interactions in histidine buffer (see Fig. 4a). However, replacing it by acetate buffer leads to an increase in *T*_½_ and *c*_½_, which also might happen due to specific interaction between acetate and HSA-NEP.

Among all tested additives, arginine has a pronounced effect on the HSA-NEP stability. At pH 5.0, it generally destabilizes, for both chemical and thermal denaturation, with an exception in the thermal denaturation in histidine with 140 mM NaCl. In general, the effect of addition of arginine seems to be comparable to the addition of NaCl for thermal denaturation. However, the chemical denaturation curves indicate that arginine may have another role in this system: helping unfolding at pH 5.0 and hampering unfolding at pH 6.5 in phosphate. Proline seems to have a clear stabilizing effect at pH 6.5 in phosphate buffer (chemical denaturation), and a clear destabilizing effect in histidine buffer +140 mM NaCl (thermal denaturation).

As already mentioned, HSA-NEP is present in different compact conformations: compact_1_, compact_2_, and compact_3_ (see Fig. 5e). For better understanding of conformational changes, the interface between HSA and NEP was studied by MD simulations. Molecular understanding about the preference of the different conformation in varying pH was explored combining the electrostatics surface study and free energy of interaction at the HSA-NEP interface.

The SAXS data shows that up to pH 7.5 only the compact_1_ and compact_2_ conformations are present. According to the analysis of the MD simulations, compact_3_ is not present below pH 7.5 due to highly unfavorable repulsive interactions in the interface region (see Fig. 7m). Moreover, *EOM* analysis shows that up to pH 7.5, the volume fraction of compact_1_ decreases, while the volume fraction for extended conformations increases (see Fig. 5c). Analysis of the MD simulations shows that compact_1_ becomes less favorable due to increasing positive charge-charge repulsion and decreasing interaction energy between HSA and NEP.

Finally, SAXS data shows that at pH 8.5 HSA-NEP is present in compact_1_, compact_3_, and extended conformations. According to MD simulation results, tris as a buffering system enhances the stability of compact_3_, binding in the interface making it more a favorable conformation along with the extended conformations (see Fig. 5c). From MD simulations at pH 8.5, compact_1_ has less pronounced charge-charge repulsion when compared to compact_2_, which means that compact_1_ is more likely to be present at pH 8.5.

**Figure 11:**
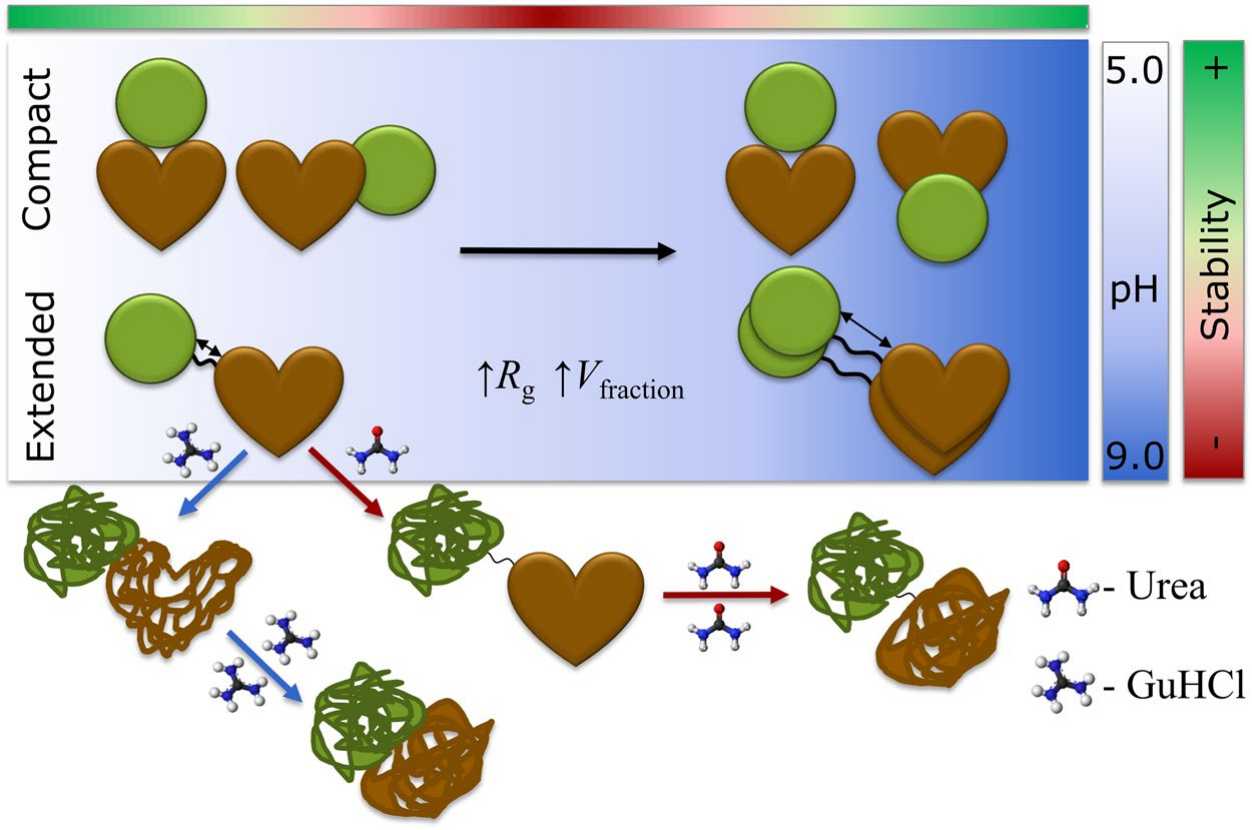
Schematic overview of HSA-NEP stability.

In combination, ICD and SAXS provide better understanding of the HSA-NEP unfolding process. HSA-NEP unfolding starts with NEP unfolding, which is followed by unfolding of HSA. Additionally, the GuHCl and urea denaturation curves point to different unfolding mechanisms (see Fig. 8). In the presence of urea, the first two transitions are well separated by well-defined intermediate state, which suggests presence of a stable intermediate, where HSA is folded and NEP is unfolded. In the presence of GuHCl, HSA-NEP also has multi-state unfolding, but the transitions between different states are not well-defined. This suggests that with GuHCl, unfolding of NEP might affect the integrity of HSA, which leads to the absence of a well-defined intermediate state (see Fig. 11).

Moreover, multiple transitions in ICD curves (see Fig. 9) can also be explained by the presence of different HSA-NEP conformations. Compact conformations have multiple interactions at the interface, which might stabilize NEP. However, in the extended conformation, the NEP domain is more exposed, resulting in a faster unfolding.

## Conclusion

From this study, we show how to explain behavior and stability of fusion proteins in general based on biophysical and molecular characterization. Our study shows that we cannot rely only on the knowledge we already have on the individual proteins. While the HSA-NEP protein shows increasing stability with decreasing repulsion, HSA alone shows the opposite^19^, as do most stable proteins. Furthermore, it is also not even possible to generalize the stability of albumin fusion proteins, as the MD simulation results clearly show the importance of specific interactions with the tris molecule indicating that for each system consisting of both protein and buffer a separate analysis is needed.

Unlike most proteins, HSA-NEP is less stable with increasing repulsion, due to intradomain repulsion. Moreover, interactions between HSA and NEP do not allow for full flexibility: HSA-NEP is present in an extended and multiple compact conformations. The compact conformations are mainly stabilized by salt bridges in the interface between HSA and NEP and are therefore more rigid, while the extended conformation is more flexible. Changes in pH induce changes at the interface, which shifts the equilibrium between different compact and extended conformations.

## Methods

### Dialysis and formulation

Dialysis and formulation procedure was performed according to the protocol described in *Kulakova et al*.^20^ HSA-NEP was provided by AstraZeneca in 49 g/L solution and was dialyzed for pH/NaCl and buffer/excipients screening. After dialysis, HSA-NEP was diluted to 20 g/L with dialysis buffer, followed by 1:20 dilution with final formulation buffer within pH of ±0.5^20^. The concentration was determined with a NanodropTM 1000 (Thermo Fisher Scientific, Waltham, USA) (see Table S.1 in supplementary information (SI)).

### Isothermal chemical denaturation

Chemical denaturation studies was on Unchained Labs HUNK system - AVIA ICD 2304 (Unchained Labs, Pleasanton, USA) according to the protocol described in *Kulakova et al*.^20^ using 1134 min of additional incubation time. Both urea and guanidine hydrochloride (GuHCl) were used as denaturants for pH/NaCl screening, while urea was selected for the buffer/excipients. Data analysis was performed using Formulator software v3.02 (Unchained Labs, Pleasanton, USA https://www.unchainedlabs.com/hunky/). For the native protein, the emission wavelength was selected from the data of the first point of the gradient, which corresponded to 336 nm. For the unfolded state, data of the last point of the gradient was considered and the maximum wavelength was 356 nm. The ratio 356/336 was plotted against denaturant concentration to monitor the unfolding process. The denaturation curve pointed to the presence of a multi-state unfolding process, therefore, both 3- and 4-state models were used for the data fitting. Additionally, secondary fits were performed for each NaCl concentration combining different pH. Free energy of unfolding (Δ*G*_unfold_), *c*_½_, and *m*-values were calculated for both transitions. HSA-NEP stability was analyzed by monitoring the change in *c*_½_ from the first transition, where the protein starts to unfold.

### Thermal denaturation

Thermal stability studies were performed in triplicate with the Prometheus NT.48 (NanoTemper Technologies, Munich, Germany) according to the protocol described in *Kulakova et al*.^20^. HSA-NEP stability was analyzed by monitoring the change in *T*_½_ from the first transition. All measurements were done in triplicates and data analysis was performed using PR.Control v1.12.2 software (NanoTemper Technologies, Munich, Germany).

### Small angle X-ray scattering

Data collection was performed at the P12 beamline at the Petra III storage ring (DESY, Hamburg DE)^21^ (see Table S.1 in SI for experimental details). Radius of gyration (*R*_g_) and maximum dimension (*D*_max_) were derived from the experimental data with the graphical data analysis program *PRIMUSQT*^22^.

*Ensemble Optimization Method* (*EOM*) was used to analyze conformational polydispersity of HSA-NEP. *EOM* consists of two programs *RANCH* and *GAJOE* that were used separately. *RANCH* was used to generate a large pool of 10000 random conformations (genes) by using HSA (pdbid: 6EZQ^16^) and NEP (pdbid: 6GID^17^) from homology modelling. Flexible regions in C-terminal of HSA (LGLG) and N-terminal of NEP (YDDGICKS) were removed from the structures, increasing the length of the linker. *GAJOE* was used to select ensembles of conformations, such that the average structure fits to the experimental data. Experimental curves acquired at different HSA-NEP concentrations were merged in order to remove the noise. Additionally, first 80 points were removed due to the presence of repulsive interactions. The output files contain fit to experimental data, distribution of volume fractions, and information about *R*_g_ and *D*_max_ distribution.

### Molecular dynamics simulations

Initially, HSA (PDB ID: 6EZQ^18^) and NEP (PDB ID: 6GID^19^) crystal structures were aligned to the most representative structures from SAXS modelling (that are shown in Fig. 5e) to get the two crystal structures in the right orientation. This structure was taken further for homology modelling to model the linker region (GGGGS) using MODELLER9.20^23^ program.

Minimization was performed on the generated models to account for structure optimization. Using PDB2PQR^24^ plugin in *PyMOL*^25^, structures were prepared at different physicochemical conditions (pH 5, 6.5, and 8.5). Subsequently, these structures were taken for all-atom classical constant pH MD simulation of 50 ns in explicit solvent utilizing ff99SB^26^ force-field for proteins. In total, the solvated system contained approximately 60000 water molecules. Each system was neutralized with either sodium or chloride depending on the overall charge of the protein. The complete protocol used to setup MD simulations is described in our previous work^27^. HSA-NEP interaction interface is defined as follows: consider HSA, residues belonging to NEP within 5 Å of HSA’s protein surface, and vice versa is defined as interface residues for HSA-NEP interaction. The interface for each of the conformations is different, hence the interface was found separately for each conformation (see Table S.6 in SI). The titratable residues such as Asp, Glu, His, Lys, in the interface were subjected to titration during constant pH MD simulations to account for change in protonation state upon protein structure dynamics. Using the MM-GBSA^28,29^ free energy method, the free energy of interaction at the interface was calculated. The most representative structure found throughout the simulation using hierarchical clustering approach^30,31^ was taken for MM-GBSA calculations. Analyses were performed with CPPTRAJ^32^ in Amber 16, and VMD 1.9.3^33^. The electrostatic surface potential was calculated for the most representative structure from the hierarchical clustering. Interface SASA was calculated by subtracting the SASA for the interface residues in HSA-NEP from the individual protein domains: HSA and NEP, respectively. All simulations were performed once for 50 ns each.

Additionally, the compact_3_ conformation was simulated independently in 10 mM and 150 mM tris to understand the effect of tris on the conformational stability. In total, 11 and 157 tris molecules corresponding to 10 mM and 150 mM tris were added to the solvated system containing approximately 60000 water molecules. The simulations were performed in duplicates for 50 ns starting froma random seed number to estimate the statistical uncertainty of the results. Tris was obtained from Zinc Database^34^. These molecules were prepared at pH 8.5 using the Ligprep tool in Schrödinger release 2016–3 (Schrödinger, LLC, New York, NY, USA)^35^. Parameter file for the tris molecule was prepared using the antechamber^36^ module in Amber 16 at pH 8.5 and applying the AM1-BCC^37^ charge method. Furthermore, an interaction score per residue (*P(I*_*score*_))^27^ was calculated to estimate the binding capacity of tris to the protein surface. The amount of tris accumulating at the HSA-NEP interaction surface was calculated by summing the number of contacts formed between the compact_3_ interface residues and tris molecules, further normalized by the number of tris molecules in the simulated system, which is defined as average normalized contact score.

The intermolecular interactions in the interface between HSA and NEP were analyzed for each compact conformation using *NCONT* from the CCP4 software^38^.

## Supplementary information

Supplementary Information.

## Data Availability

ICD, nanoDSF and SAXS data is deposited in PIPPI Data Base https://pippi-data.kemi.dtu.dk/ *EOM* models obtained from SAXS data analysis can be found in SASBDB database.
