# Supplementary material for: Albumin-neprilysin fusion protein: understanding stability using small angle X-ray scattering and molecular dynamic simulations
Source: Sci Rep. 2020 Jun 22;10:10089. doi: 10.1038/s41598-020-67002-9 (PMC7308280; doi:10.1038/s41598-020-67002-9)
Supplement: Supplementary file 1 — Supplementary Information. [file 41598_2020_67002_MOESM1_ESM.pdf]

Supplementary material

Albumin-neprilysin fusion protein: understanding stability using small angle X-ray scattering and molecular dynamic simulations

Alina Kulakova[1], Sowmya Indrakumar[1], Pernille Sønderby[1], Sujata Mahapatra[2], Werner W. Streicher[2], Günther H.J. Peters[1], Pernille Harris[1]

[1]Department of Chemistry, Technical University of Denmark, 2800 Kgs. Lyngby, Denmark

[2]Novozymes A/S, Biologiens Vej 2, 2800 Kongens Lyngby, Denmark

Table S.1: SAXS experimental details

a) Sample details			
	HSA-NEP fusion protein		
Organism	Chinese Hamster Ovary (CHO) cells		
Source	AstraZeneca		
Extinction coefficient (Abs 0.1% = 1 g/L)	1.04		
Molecular weight *MW* from chemical composition (kDa)	146.7		
b) SAXS data collection			
Instrument	P12 BioSAXS beamline (PETRAIII)		
Date	12.17	07.18	12.18
Detector	PILATUS2M	PILATUS6M	
Wavelength (nm)	1.240	1.244	
Beam size (mm^2)	0.2 × 0.12		
Detector distance (m)	3.000		
q-measurement range (nm^{-1})	0.027-5.078	0.026 -7.288	0.0261-7.263
Absolute scaling method	Comparison with scattering from pure H_2O		Comparison with scattering from BSA
Normalization	To transmitted intensity by beam-stop counter		
Monitoring for radiation damage	Frame-by-frame comparison		
Exposure time (s)	20 x 0.05		30 x 0.095
Sample configuration	Quartz glass capillary		
Sample temperature (°C)	20		
c) Software employed for SAXS data reduction, analysis and interpretation			
SAS data reduction	*PRIMUSqt* [1] from *ATSAS* 2.8.3[2]		
Basic analyses: Guinier, $p(r)$, V_P	*PRIMUSqt* [1]		
Ensemble representation of atomic models	*EOM* [3,4]		
Molecular graphics	*PyMOL* (version 1.8.2.3, Schrödinger, LLC)		
Figures	*MatLab*		

Table S.2: SAXS data collection overview

Buffer	pH	Additives	$c_{additives}$ (M)	$c_{HSA\text{-}NEP}$ (g/L)
10 mM histidine	5.0	-	-	1.05, 1.62, 3.72, 5.39, 7.42, 10.12, and 14.09
		NaCl	0, 0.035, 0.070, and 0.140	5.83, 6.06, 6.08, and 5.70
	5.5	-	-	1.20, 2.03, 4.76, 6.62, 9.12, 12.45, and 16.66
		Urea	0, 1, 1.5, 3, 5.5, and 8	6.15, 5.39, 5.7, 5.85, 5.73, and 5.56
	6.5	-	-	1.77, 3.50, 8.74, 12.07, and 17.30
		NaCl	0, 0.035, 0.070, and 0.140	6.05, 5.83, 5.69, and 5.44
		Arginine	0, 0.035, 0.070, and 0.140	2.80, 2.11. 2.20, and 1.82
	7.5	-	-	1.16, 2.40, 5.65, 8.11, 11.30, 17.02, and 22.69
10 mM phosphate	6.5	-	-	2.09, 4.11, 9.59, 15.26, and 20.52
10 mM tris	8.5	-	-	1.02, 2.29, 5.49, 7.56, and 10.49

Table S.3: Structural parameters derived from SAXS experiments

	$c_{HSA\text{-}NEP}$ (g/L)	c_{NaCl} (mM)	$c_{arginine}$ (mM)	Guinier		p(r)			Apparent MW (kDa)	
				$I(0)/c$	R_g (nm)	$I(0)/c$	R_g (nm)	D_{max} (nm)	Guinier	$p(r)$
10 mM histidine pH 5.0	1.05	-	-	0.13	4.91	0.13	5.07	16.00	180	180
	1.62	-	-	0.13	4.88	0.13	5.06	16.30	180	180
	3.72	-	-	0.13	4.77	0.13	4.97	16.16	180	180
	5.39	-	-	0.13	4.70	0.13	4.91	15.60	180	180
	7.42	-	-	0.13	4.63	0.13	4.88	15.10	180	180
	10.12	-	-	0.13	4.56	0.13	4.83	15.00	180	180
	14.09	-	-	0.13	4.51	0.13	4.81	14.90	180	180
	5.83	0	-	23808*	5.03	23990*	5.19	17.72	159	160
	6.06	35	-	23961*	5.14	24030*	5.25	17.70	160	160
	6.08	70	-	23662*	5.05	23950*	5.25	18.00	158	160
	5.70	140	-	23984*	5.19	24010*	5.29	17.50	160	160
10 mM histidine pH 5.5	1.20	-	-	0.11	4.86	0.11	5.05	15.60	152	152
	2.03	-	-	0.10	4.78	0.11	4.98	15.40	138	152
	4.76	-	-	0.11	4.76	0.11	5.08	15.70	152	152
	6.62	-	-	0.10	4.45	0.10	4.83	15.10	133	138
	9.12	-	-	0.09	4.33	0.10	4.75	15.10	129	138
	12.45	-	-	0.09	4.16	0.10	4.69	15.00	123	138
	16.66	-	-	0.08	3.68	0.09	4.45	14.50	107	125
10 mM histidine pH 6.5	1.77	-	-	0.13	4.66	0.13	5.01	15.70	180	180
	3.50	-	-	0.12	4.57	0.13	4.95	15.70	166	180
	8.74	-	-	-	-	0.12	4.90	15.50	-	166
	12.07	-	-	-	-	0.11	4.89	15.40	-	152
	17.30	-	-	-	-	0.10	4.67	15.10	-	138
	6.05	0	-	20350*	4.14	22620*	4.85	15.10	136	151
	5.83	35	-	21725*	4.65	22110*	4.89	15.50	145	148
	5.69	70	-	22255*	4.76	22540*	4.96	15.70	149	150
	5.44	140	-	22498*	4.79	22830*	5.00	15.80	150	152
	2.80	-	0	0.086	4.62	0.09	4.95	15.77	119	125
	2.11	-	35	0.093	5.00	0.09	5.13	15.80	129	125
	2.20	-	70	0.093	5.03	0.09	5.17	16.00	129	125
	1.82	-	140	0.090	5.17	0.09	5.21	16.20	125	125
10 mM phosphate pH 6.5	2.09	-	-	0.12	4.87	0.12	5.07	15.50	166	166
	4.11	-	-	0.11	4.59	0.11	4.90	15.50	152	152
	9.59	-	-	0.10	4.14	0.10	4.52	14.40	133	138
	15.26	-	-	0.09	3.95	0.09	4.33	14.20	125	125
	20.52	-	-	0.08	3.60	0.08	4.26	14.20	111	111
10 mM histidine pH 7.5	1.16	-	-	0.12	5.03	0.12	5.45	16.60	166	166
	2.40	-	-	0.12	4.92	0.12	5.43	16.60	166	166
	5.65	-	-	0.12	4.98	0.13	5.50	17.00	166	180
	8.11	-	-	0.12	4.92	0.13	5.42	17.00	166	180
	11.30	-	-	0.12	4.76	0.13	5.53	17.80	166	180
	17.02	-	-	-	-	0.13	5.22	16.60	-	180
	22.69	-	-	-	-	0.13	5.11	16.00	-	180
10 mM tris pH 8.5	1.02	-	-	23795*	4.92	24490*	5.28	16.80	159	163
	2.29	-	-	22704*	4.70	24020*	5.18	16.20	152	160
	5.49	-	-	20674*	4.30	23280*	5.05	16.00	138	155
*Note: * BSA calibration*	7.56	-	-	-	-	24800*	5.21	16.50	-	166
	10.49	-	-	-	-	24350*	5.14	16.50	-	163

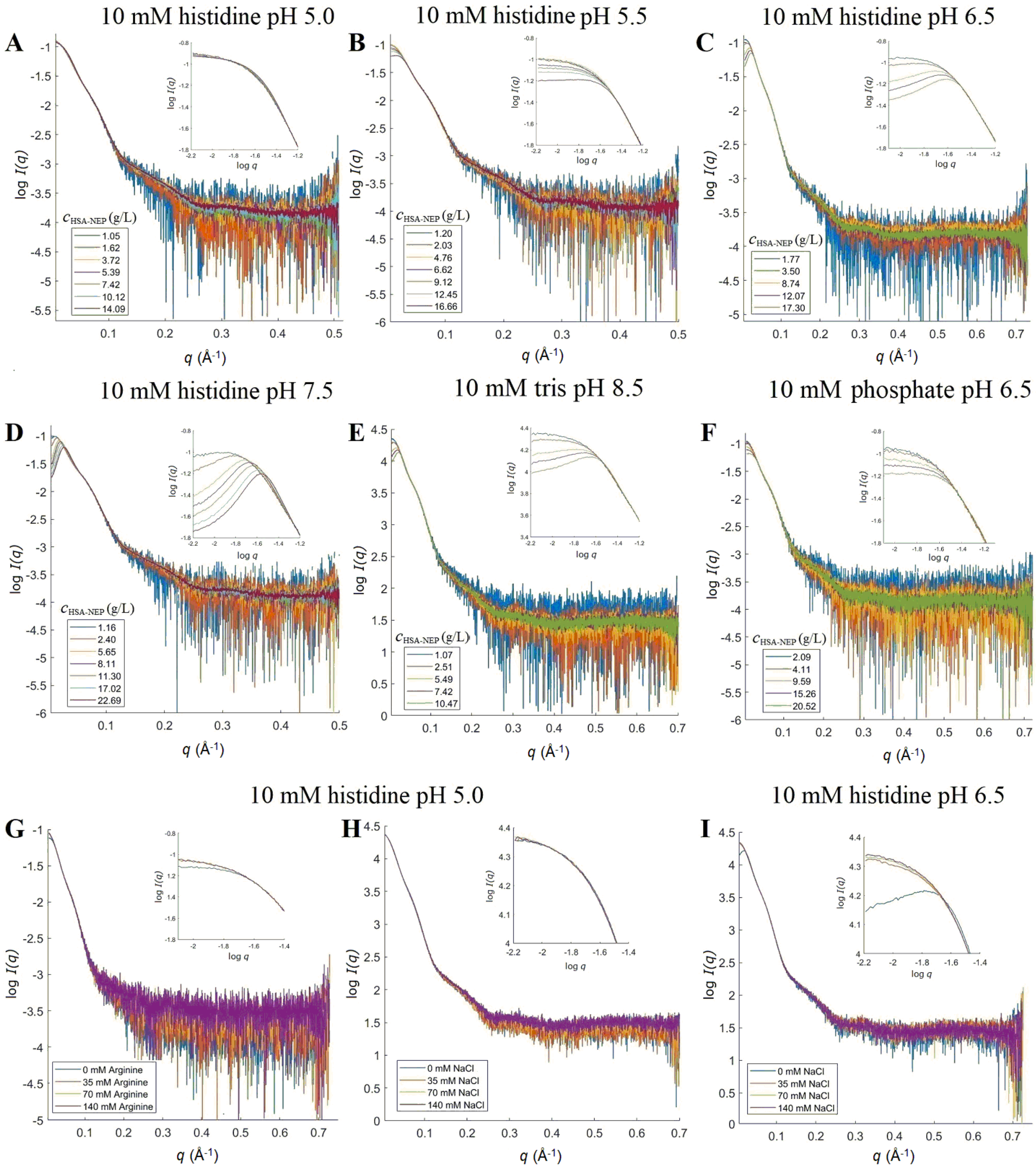

Figure S.1: SAXS data collected at different pH with and without additives. Concentration series: A: 10 mM histidine pH 5.0; B: 10 mM histidine pH 5.5; C: 10 mM histidine pH 6.5; D: 10 mM histidine pH 7.5; E: 10 mM tris pH 8.5; F: 10 mM phosphate pH 6.5. Additives: 10 mM histidine pH 5.0 with G: arginine with $c_{\text{HSA-NEP}}$ around 2 g/L and H: NaCl with $c_{\text{HSA-NEP}}$ around 5.5-6 g/L; I: 10 mM histidine pH 6.5 with NaCl with $c_{\text{HSA-NEP}}$ around 6 g/L.

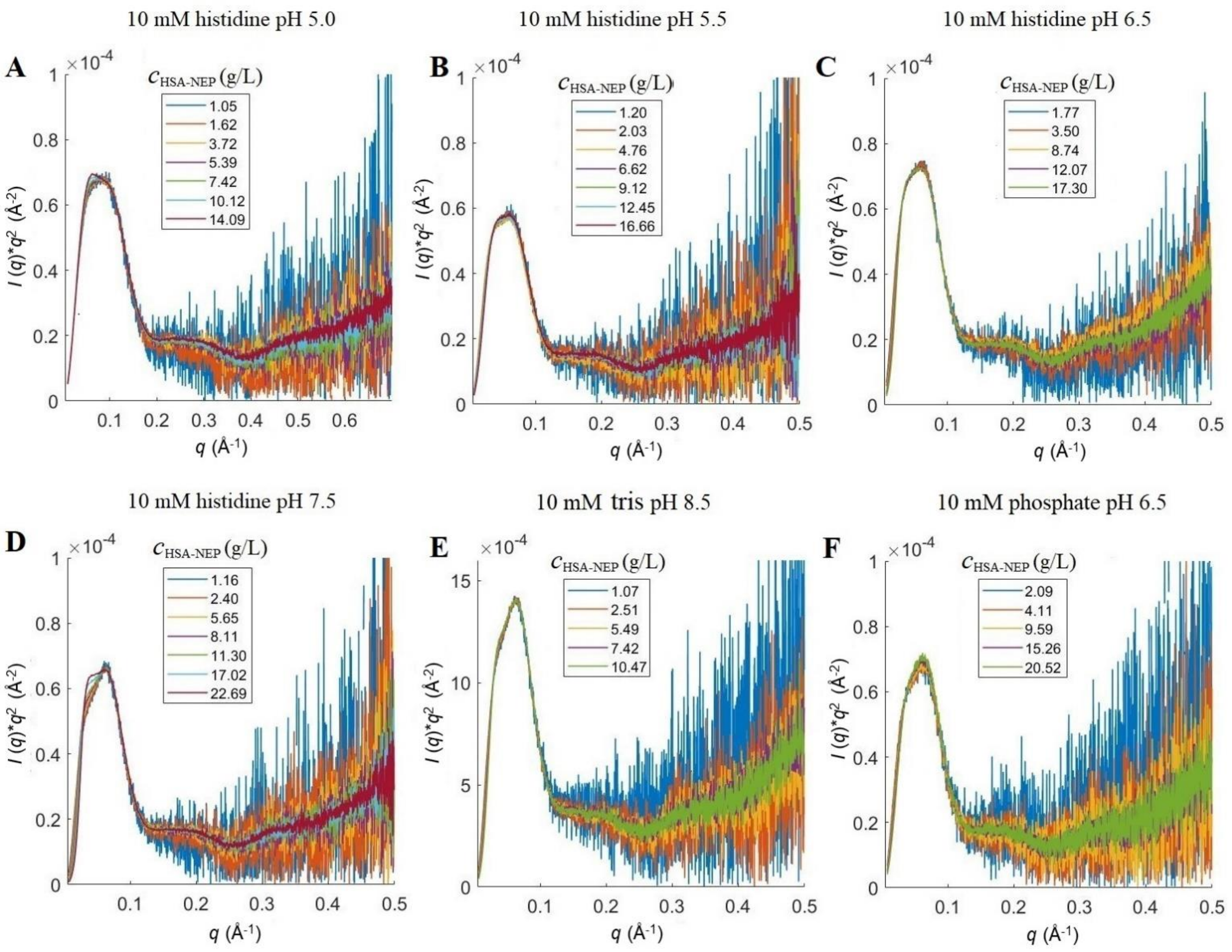

Figure S.2: Kratky plot of a HSA-NEP at different pH: A: 10 mM histidine at pH 5.0; B: 10 mM histidine at pH 5.5; C: 10 mM histidine at pH 6.5; D: 10 mM histidine at pH 7.5; E: 10 mM tris at pH 8.5; F: 10 mM phosphate at pH 6.5.

Table S.4: Structural parameters for merged files used for *EOM*

	**Histidine**				**Tris**	**Phosphate**
	**pH 5.0**	**pH 5.5**	**pH 6.5**	**pH 7.5**	**pH 8.5**	**pH 6.5**
**Guinier analysis**						
$I(0)/c$ ($Lg^{-1}cm^{-1}$)	0.11	0.09	0.11	0.10	20250*	0.10
R_g (nm)	4.38	4.33	4.28	4.36	4.35	4.27
q_{min} (nm^{-1})	0.2812	0.2839	0.2964	0.2875	0.2802	0.2964
MW from *I*(0) (ratio to predicted) (kDa)	152 (1.04)	127 (0.86)	152 (1.04)	133 (0.91)	135 (0.92)	138 (0.94)
***p*(*r*) analysis**						
$I(0)$ (cm^{-1})	0.12	0.10	0.13	0.11	24520*	0.12
R_g (nm)	4.83	4.93	5.05	5.15	5.28	5.06
D_{max} (nm)	15.00	15.30	16.00	16.80	16.40	16.00
q range (nm^{-1})	0.2812-1.9013	0.2867-1.9013	0.2964-2.0127	0.2802-1.9058	0.2930-1.9082	0.2964-1.9989
χ^2 (total estimate from *GNOM*)	2.897	2.650	3.622	2.908	3.083	1.395
MW from *I*(0) (ratio to predicted) (kDa)	166 (1.13)	138 (0.94)	180 (1.23)	152 (1.04)	164 (1.12)	166 (1.13)
Porod volume (nm^{-3}) (ratio V_p/calculated *MW*)	235.2 (1.70)	243.7 (1.70)	245.0 (1.70)	241.4 (1.70)	245.03 (1.70)	236.43 (1.70)
V, *MW* using Fischer method (ratio of *MW* to expected)	138.4 (0.94)	143.4 (0.98)	144.1 (0.98)	142.0 (0.97)	144.1 (0.98)	139.1 (0.95)

Table S.5: R_g and D_{max} for each HSA-NEP conformation.

**Conformation**	R_g **(nm)**	D_{max} **(nm)**
$Compact_1$	3.8	13.5
$Compact_2$	4.3	14.8
$Compact_3$	3.8	12.5
Extended	5.1-5.5	15.7-17.5

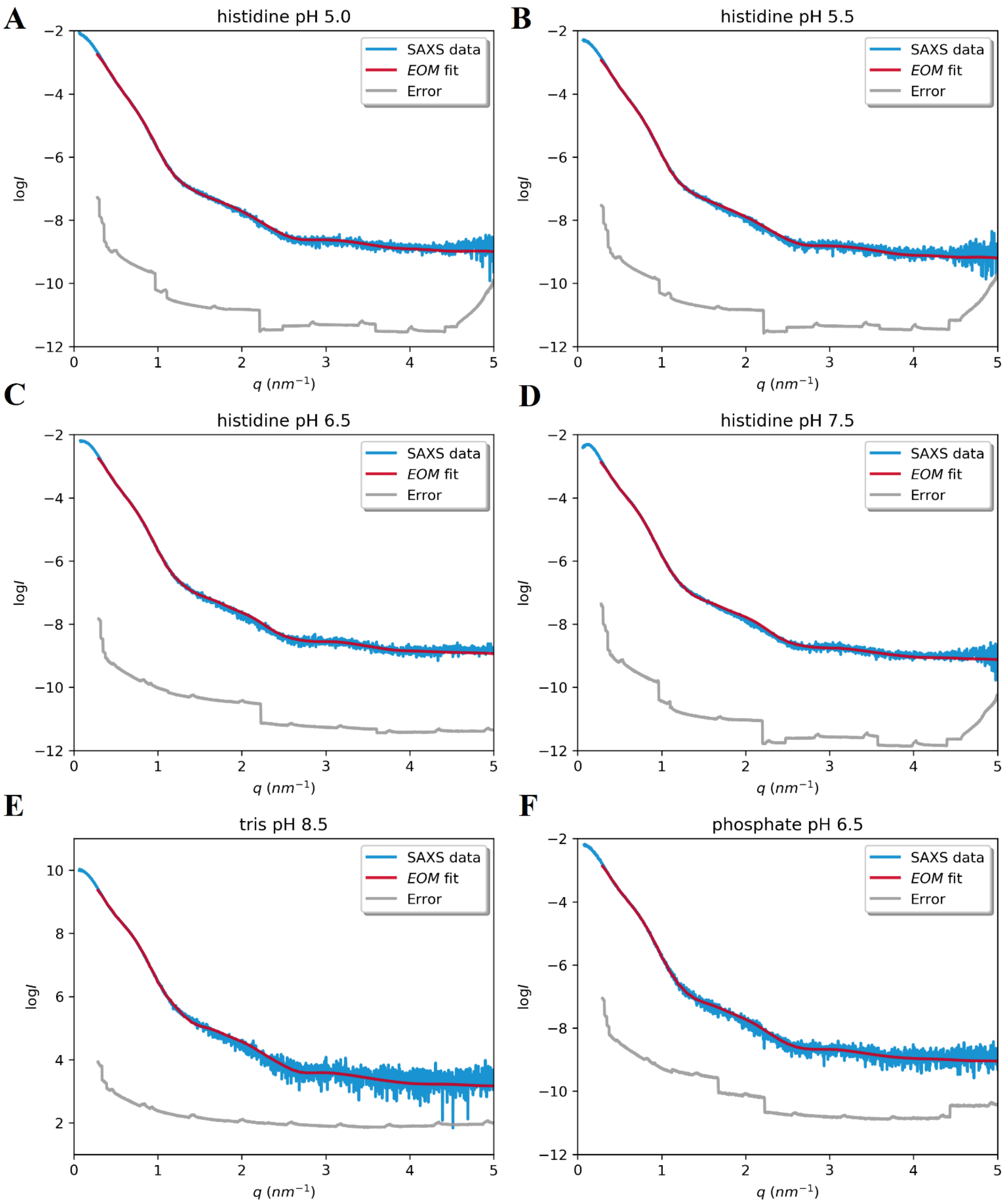

Figure S.3: Fit of *EOM* results against merged experimental data. A: 10 mM histidine pH 5.0; B: 10 mM histidine pH 5.5; C: 10 mM histidine pH 6.5; D: 10 mM histidine pH 7.5; E: 10 mM tris pH 8.5; F: 10 mM phosphate pH 6.5.

Table S.6: Residues that form the HSA-NEP interaction interface for each of the four conformation are listed.

Conformation	HSA interface residue	Linker	NEP interface residue
Extended	499, 500-506, 535-564, 566-585	586-590	591-608, 611, 612, 628-630, 636-674, 677-681, 683-687, 946-948, 966, 969-972,1106-1108
$Compact_1$	113-125, 173, 419-422, 483, 466, 467, 498-517, 527, 535, 536, 538, 558-569, 576-585	586-590	591, 592, 597-611, 615, 629, 630, 632-639, 648-665, 924, 925, 928-936, 939-948, 966, 969, 970, 1101, 1171, 1172, 1215, 1216, 1238, 1239
$Compact_2$	397, 398, 538-550, 553, 557-585	586-590	659-669, 672, 673, 676, 677, 680, 1042-1044
$Compact_3$	471, 472, 475, 479, 490-492, 494-502, 535-539, 580-585	586-590	591, 598- 600, 602-607, 632-636, 1215, 1216

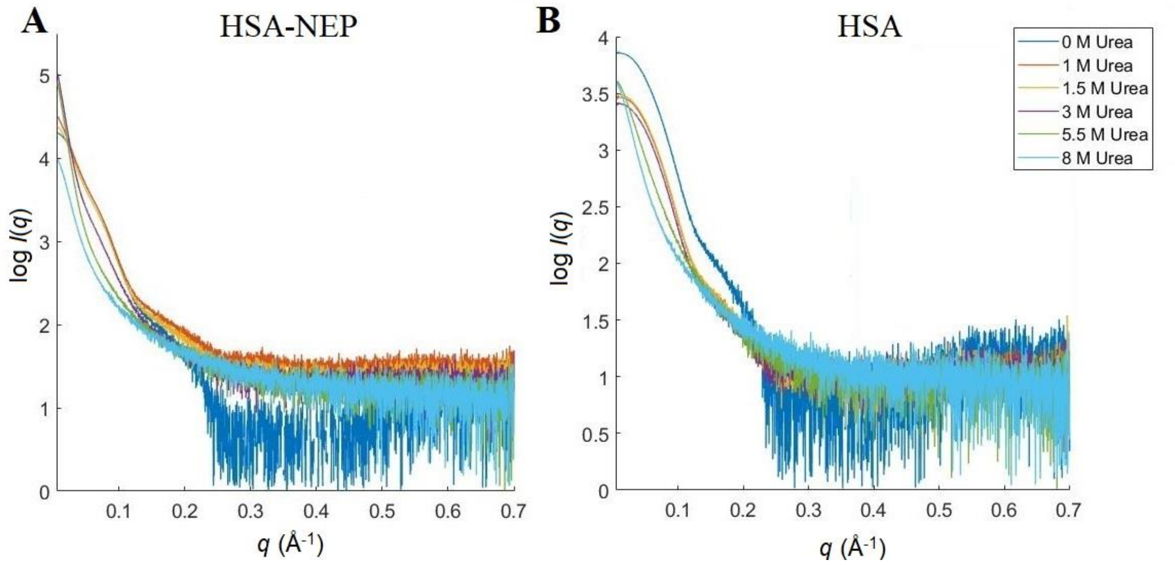

Figure S.4: SAXS scattering curves for A: HSA-NEP and B: HSA in 10 mM histidine pH 5.5 at different concentrations of urea with $c_{\text{HSA-NEP}}$ around 5 g/L.

Sequence

DAHKSEVAHRFKDLGEENFKALVLIAFAQYLQQSPFEDHVKLVNEVTEFAKTCVADESAENCDKSLHTLFGDKLCT
VATLRETYGEMADCCAKQEPERNECFLQHKDDNPNLPRLVRPEVDVMCTAFHDNEETFLKKYLYEIARRHPYFYA
PELLFFAKRYKAAFTECCQAADKAACLLPKLDELRDEGKASSAKQRLKCASLQKFGERAFKAWAVARLSQRFPKA
EFAEVSKLVTDLTKVHTECCHGDLLECADDRADLAKYICENQDSISSKLKECCEKPLLEKSHCIAEVENDEMPADLP
SLAADFVESKDVCKNYAEAKDVFLGMFLYEYARRHPDYSVVLLLRLAKTYETTLEKCCAAADPHECYAKVFDEFK
PLVEEPQNLIKQNCELFEQLGEYKFQNALLVRYTKKVPQVSTPTLVEVSRNLGKVGSKCCKHPEAKRMPCAEDYLS
VVLNQLCVLHEKTPVSDRVTKCCTESLVNRRPCFSALEVDETYVPKEFNAETFTFHADICTLSEKERQIKKQTALVE
LVKHKPKATKEQLKAVMDDFAAFVEKCCKADDKETCFAEEGKKLVAASQAALGL**GGGGS**YDDGICKSSDCIKSA
ARLIQNMDATTEPCTDFFKYACGGWLKRNVIPETSSRYGNFDILRDELEVVLKDVLQEPKTEDIVAVQKAKALYRS
CINESAIDSRGGEPLLKLLPDIYGWPVATENWEQKYGASWTAEKAIAQLNSKYGKKVLINLFVGTDDKNSVNHVIHI
DQPRLGLPSRDYYECTGIYKEACTAYVDFMISVARLIRQEERLPIDENQLALEMNKVMELEKEIANATAKPEDRNDP
MLLYNKMTLAQIQNNFSLEINGKPFSWLNFTNEIMSTVNISITNEEDVVVYAPEYLTKLKPILTKYSARDLQNLMSW
RFIMDLVSSLSRTYKESRNAFRKALYVTTSETATWRRCANYVNGNMENAVGRLYVEAAFAGESKHVVEDLIAQIRE
VFIQTLDDLTWMDAETKKRAEEKALAIKERIGYPDDIVSNDNKLNNEYLELNYKEDEYFENIIQNLKFSQSKQLKKL
REKVDKDEWISGAAVVNAFYSSGRNQIVFPAGILQPPFFSAQQSNSLNYGGIGMVIGHEITHGFDDNGRNFNKDGDL

VDWWTQQSASNFKEQSQCMVYQYGNFSWDLAGGQHLNGINTLGENIADNGGLGQAYRAYQNYIKKNGEEKLLP
GLDLNHKQLFFLNFAQVWCGTYRPEYAVNSIKTDVHSPKNFRIIGTLQNSAEFSEAFHCRKNSYMNPEKKCRVW
Linker

References

(1) Konarev, P. V.; Volkov, V. V.; Sokolova, A. V.; Koch, M. H. J.; Svergun, D. I. PRIMUS: A Windows PC-Based System for Small-Angle Scattering Data Analysis. *J. Appl. Crystallogr.* **2003**, *36* (5), 1277–1282. https://doi.org/10.1107/S0021889803012779.

(2) Franke, D.; Petoukhov, M. V.; Konarev, P. V.; Panjkovich, A.; Tuukkanen, A.; Mertens, H. D. T.; Kikhney, A. G.; Hajizadeh, N. R.; Franklin, J. M.; Jeffries, C. M.; et al. ATSAS 2.8 : A Comprehensive Data Analysis Suite for Small-Angle Scattering from Macromolecular Solutions. *J. Appl. Crystallogr.* **2017**, *50* (4), 1212–1225. https://doi.org/10.1107/S1600576717007786.

(3) Tria, G.; Mertens, H. D. T.; Kachala, M.; Svergun, D. I. Advanced Ensemble Modelling of Flexible Macromolecules Using X-Ray Solution Scattering. *IUCrJ* **2015**, *2*, 207–217. https://doi.org/10.1107/S205225251500202X.

(4) Bernadó, P.; Mylonas, E.; Petoukhov, M. V.; Blackledge, M.; Svergun, D. I. Structural Characterization of Flexible Proteins Using Small-Angle X-Ray Scattering. *J. Am. Chem. Soc.* **2007**, *129* (17), 5656–5664. https://doi.org/10.1021/ja069124n.
